# Evaluation of elderly specific pre-hospital trauma triage criteria: a systematic review

**DOI:** 10.1186/s13049-021-00940-z

**Published:** 2021-08-30

**Authors:** Adam J. Boulton, Donna Peel, Usama Rahman, Elaine Cole

**Affiliations:** 1 Academic Department of Anaesthesia, Critical Care, Pain and Resuscitation, Heartlands Hospital, University Hospitals Birmingham NHS Foundation Trust, Birmingham, B9 5SS UK; 2 Warwick Medical School, University of Warwick, Coventry, UK; 3 Brighton & Sussex University Hospitals NHS Trust, Brighton, UK; 4 Centre for Trauma Sciences, Blizard Institute, Barts and The London School of Medicine and Dentistry, Queen Mary University of London, London, UK

**Keywords:** Elderly trauma, Geriatric trauma, Pre-hospital triage, Elderly triage, Pre-hospital elderly trauma, Emergency medical services

## Abstract

**Background:**

Pre-hospital identification of major trauma in elderly patients is key for delivery of optimal care, however triage of this group is challenging. Elderly-specific triage criteria may be valuable. This systematic review aimed to summarise the published pre-hospital elderly-specific trauma triage tools and evaluate their sensitivity and specificity and associated clinical outcomes.

**Methods:**

MEDLINE and EMBASE databases were searched using predetermined criteria (PROSPERO: CRD42019140879). Two authors independently assessed search results, performed data extraction, risk of bias and quality assessments following the Grading of Recommendations, Assessment, Development and Evaluation system.

**Results:**

801 articles were screened and 11 studies met eligibility criteria, including 1,332,300 patients from exclusively USA populations. There were eight unique elderly-specific triage criteria reported. Most studies retrospectively applied criteria to trauma databases, with few reporting real-world application. The Ohio Geriatric Triage Criteria was reported in three studies. Age cut-off ranged from 55 to 70 years with ≥ 65 most frequently reported. All reported existing adult criteria with modified physiological parameters using higher thresholds for systolic blood pressure and Glasgow coma scale, although the values used varied. Three criteria added co-morbidity or anti-coagulant/anti-platelet use considerations. Modifications to anatomical or mechanism of injury factors were used by only one triage criteria. Criteria sensitivity ranged from 44 to 93%, with a median of 86.3%, whilst specificity was generally poor (median 54%). Scant real-world data showed an increase in patients meeting triage criteria, but minimal changes to patient transport destination and mortality. All studies were at risk of bias and assessed of “very low” or “low” quality.

**Conclusions:**

There are several published elderly-specific pre-hospital trauma triage tools in clinical practice, all developed and employed in the USA. Consensus exists for higher thresholds for physiological parameters, however there was variability in age-cut offs, triage criteria content, and tool sensitivity and specificity. Although sensitivity was improved over corresponding ‘adult’ criteria, specificity remained poor. There is a paucity of published real-world data examining the effect on patient care and clinical outcomes of elderly-specific triage criteria. There is uncertainty over the optimal elderly triage tool and further study is required to better inform practice and improve patient outcomes.

**Supplementary Information:**

The online version contains supplementary material available at 10.1186/s13049-021-00940-z.

## Introduction

The proportion of elderly people experiencing major trauma is increasing as healthcare systems globally cope with an ageing population [1–5]. Despite advances in trauma management the elderly population has worse outcomes compared to younger adults with similar injury severity [6, 7]. Fewer elderly trauma patients are initially triaged to major trauma centres (Level 1 equivalent centres), are less likely to trigger trauma team activation, and more likely to be initially assessed by junior doctors [5]. Early and accurate identification of the elderly major trauma patient is essential to guide appropriate care pathways [8, 9]. Improvements in the triage of elderly trauma patients have the potential to significantly improve clinical outcomes, whilst also being resource efficient [5, 8].

Current pre-hospital triage tools are ineffective in identifying elderly major trauma patients [5, 10–13]. Developing a pre-hospital triage tool with acceptable under- and over-triage rates, whilst also being useable in the pre-hospital environment, is challenging. Field triage tools use a combination of vital signs, and anatomy and mechanism of injury factors [14]. The physiological response to trauma alters with age and vital signs may not change as in younger patients [11, 15, 16]. Additionally, co-morbidities and concurrent medications may make presentation of major trauma less obvious in elderly patients and mask underlying instability. The traditional paradigm of major trauma triage emphasises identification of high-energy transfer mechanisms [17]. However, in elderly patients major trauma frequently occurs after lower energy transfer mechanisms [4, 5]. Falls have become the greatest single cause of trauma in the USA, accounting for almost half of trauma mechanisms [1]. A fall from < 2 m height has been the most common injury mechanism for major trauma in the UK since 2008 with its proportion continually growing [4, 5]. Globally, falls have been identified as a major public health concern and the WHO report that falls are the second leading cause of unintentional injury-related death [18]. These challenges result in high levels of under-triage of elderly trauma patients and the inability to quickly and effectively direct ongoing care [5, 13]. Considering the mechanistic and physiological differences, major trauma triage tools specifically developed for the elderly patient population may be more efficacious. Such triage tools must apply to all pre-hospital elderly trauma patients if this high under-triage rate is to be improved [5].

There is currently no national or global consensus of how best to identify elderly patients with major traumatic injuries in the pre-hospital environment. There is a timely need to identify an effective pre-hospital elderly major trauma triage tool to optimise care delivery and improve mortality and morbidity. This systematic review aimed to summarise the published elderly-specific criteria for pre-hospital triage following trauma, evaluate criteria sensitivity and specificity, and examine the associated clinical outcomes.

## Methods

This systematic review was prospectively registered on PROSPERO (Reference: CRD42019140879) and the PRISMA guidelines were followed in its conduct and reporting [19].

### Research questions

The systematic review sought to investigate the following research questions:

- Which elderly-specific criteria are included in pre-hospital trauma triage tools?
- What is the reported sensitivity and specificity of elderly-specific trauma triage criteria used within the pre-hospital setting?
- What are the associations between pre-hospital elderly-specific trauma triage criteria and clinical outcomes?

### Eligibility criteria

Eligibility was determined using PICO [20]. The population was elderly patients with traumatic injuries. Our initial scoping reviews revealed a wide range of age-cut-offs and therefore a conservative age cut-off of ≥ 50 years was used to ensure all relevant studies were captured. The intervention was the development or implementation of elderly-specific triage criteria. No specific comparators were predefined. The primary outcome was criteria sensitivity and specificity. Secondary outcomes were pre-defined clinical outcomes as detailed below. Original articles written in English were included. Conference abstracts and abstract-only articles where full text was not available were excluded, as were preclinical/animal studies.

### Search strategy

The search strategy utilised OVID to search MEDLINE from 1946 and MEDLINE In-Process & Other Non-Indexed Citations and EMBASE from 1974. The search terms were a combination of keywords and MeSH terms: “trauma”, “Wounds & Injuries”, “Emergency Medical Services”, “pre-hospital care” “Triage”, “Geriatrics”, “older adults”, “elderly”, “Humans”. These were combined with operators OR and AND to expand and then refine the search. An example of the search is shown in Additional File 1.

### Study selection

Search results were independently reviewed by two authors (AJB and DP). Titles and abstracts of all returned articles were screened for eligibility and full texts obtained for those meeting eligibility criteria. The reference lists of included studies were screened in the same manner. All duplicate studies were removed. Any discrepancies were discussed and where consensus could not be reached, this was resolved with a third author (UR).

### Data extraction and analysis

Two authors (AJB and DP) independently extracted data using predetermined criteria. Study-level data included author and publication year, study design, country of origin, eligibility criteria, and description of elderly-specific triage criteria. Patient-level data included demographics (age and gender), injury severity, and mechanism of injury (MOI). The outcome measures to be extracted were sensitivity and specificity by Injury Severity Score (ISS) > 15 and ISS > 9, in-hospital mortality, length of critical care stay, length of hospital stay, time to theatre, life-saving interventions, interhospital transfers, and reported compliance with triage criteria. Pooling of studies was not appropriate due to the high methodological and clinical heterogeneity.

### Assessment of risk of bias and quality of evidence

Risk of bias was assessed using the Newcastle–Ottawa scale [21]. The quality of the evidence of included studies was assessed using the Grading of Recommendations, Assessment, Development, and Evaluation (GRADE) methodology [22].

## Results

### Search results

**Fig. 1:**
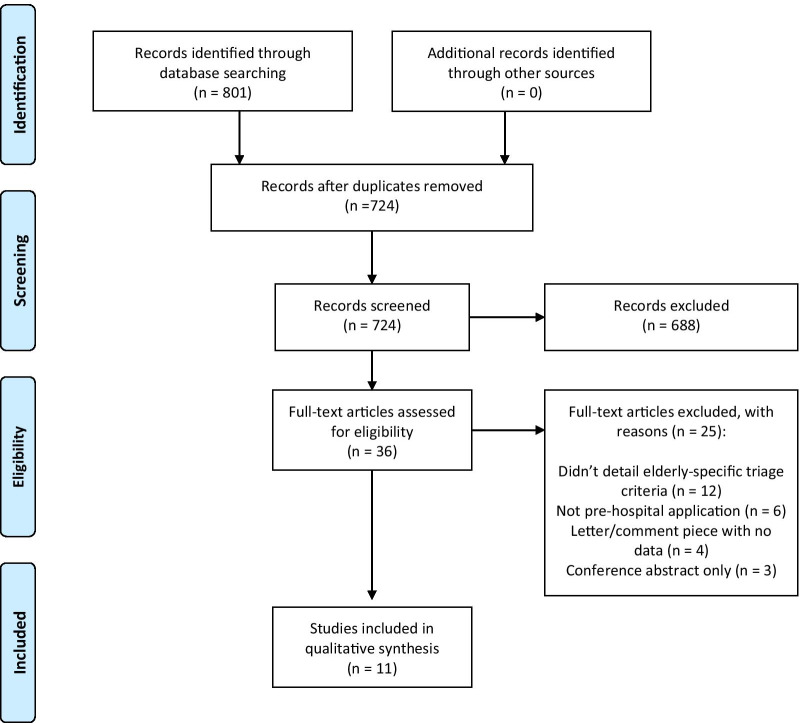
PRISMA flow diagram of search results. PRISMA, Preferred Reporting Items for Systematic Reviews and Meta-Analyses

The search identified 801 articles, and after duplicates were removed, 724 articles remained. After title and abstract screening, 36 articles were potentially eligible for inclusion and full texts obtained. Of these, 25 articles were excluded after full text review, 11 studies met all eligibility criteria and were included for analysis (Fig. 1) [23–33]. No additional studies were identified from reference list screening.

### Study characteristics

Study characteristics are summarised in Table 1. All studies were conducted in the USA. All studies were observational and retrospective in design utilising data from established trauma databases, except for one which used a consensus panel of experts to develop elderly triage criteria [32]. The majority of studies (n = 8) retrospectively applied triage criteria to test triage criteria efficacy [23, 25–30, 33]. Two studies tested real-world application of triage criteria using a before and after design with retrospective analysis of efficacy [24, 31].

**Table 1:** Study characteristics

References	Design	Country	Real life application or application to a database?	Database used	Derived criteria in study?	Elderly definition
Brown et al. [23]	Observational, retrospective	USA	Criteria applied to database	National Trauma Data Bank	No. substituted SBP < 110 instead of < 90 in 2011 ACS-COT Field Triage Scheme	> 65 years
Caterino et al. [24]	Observational, retrospective. Interrupted time series analysis	USA	Real life. Before and after	Ohio trauma registry	No. before and after study. 3 years before Ohio Geriatric Trauma Triage Criteria adoption and 3 years after	> 70 years
Cull et al. [25]	Observational, retrospective	USA	Criteria derived and applied to database	National Trauma Data Bank	Yes. Binary regression equations	≥ 65 years
Ichwan et al. [26]	Observational, retrospective	USA	Criteria applied to database	Ohio trauma registry	No. Applied Ohio Geriatric Trauma Triage Criteria to database	≥ 65 years
Newgard et al. [27]	Observational, retrospective	USA	Criteria applied to database	Trauma registries and ED and discharge databases linked to EMS records. 44 EMS agencies to 51 hospitals in 7 regions	No. Applied geriatric triage criteria to database	≥ 65 years
Newgard et al. [28]	Observational, retrospective	USA	Criteria derived and applied to database	Trauma registries and ED and discharge databases linked to EMS records. 94 EMS agencies to 122 hospitals in 7 regions	Yes. Classification and regression tree (CART) analysis	≥ 65 years
Newgard et al. [28]	Observational, retrospective	USA	Criteria derived and applied to database	Trauma registries and ED and discharge databases linked to EMS records. 94 EMS agencies to 122 hospitals in 7 regions	Yes. Classification and regression tree (CART) analysis	≥ 55 years
Nishijima et al. [30]	Observational, retrospective	USA	Criteria applied to database	Northern California, 5 EMS agencies transport patients to 11 general acute care hospitals	No	≥ 55 years
Scheetz [31]	Observational, retrospective. Interrupted time series analysis	USA	Real life. Before and after	National Automotive Sampling System Crashworthiness Data System	No	≥ 55 years
Wasserman et al. [32]	Consensus panel of specialist experts	USA	Consensus panel	9 experts- EMS, Pre-hospital, TBI, geriatric medicine, trauma surgery geriatric medicine	Yes, by expert panel	≥ 55 years
Werman et al. [33]	Literature search and observational retrospective	USA	Criteria derived from database	Ohio trauma registry	Yes, identified by literature search, derived from database	≥ 70 years

Three studies utilised the Ohio Trauma Registry [24, 26, 33], two accessed data from the National Trauma Data Bank [23, 25], and one used the National Automotive Sampling Crashworthiness System Database [31]. A further four studies [27–30] used local state or system-wide databases and with the exception of one [32], all studies were multi-centred.

Study inclusion and exclusion criteria varied, but typically included patients with traumatic injury transported by Emergency Medical Services (EMS) to hospital [24, 26–31], excluding inter-hospital transfers [23, 28–30], isolated hip fractures [24, 26, 33], and death before hospital arrival [23, 28, 29]. Two studies received grants from the Ohio Department of Public Safety [24, 26], two from the Robert Wood Johnson Foundation [28, 29], one from the Centre for Disease Control [30], one from the Agency for Healthcare Research and Quality [27], and one from local university funding [25]. Three studies did not report a funding source [31–33] and one study stated it did not receive any funding [23].

### Patient characteristics

There were 1,332,300 included patients (Table 2). The definition of an elderly patient was variable, with four using greater than 55 years [29–32], five using 65 years [23, 25, 27, 28] and two using 70 years [24, 26]. The average age of included patients ranged from 65 to 82 years [26, 31]. Females were more commonly represented in seven studies [23, 24, 26–29, 31], one study had a male majority [30] and two did not report patient demographics [25, 33]. Where MOI was reported (n = 9) [23–31], the most common causes were reported as blunt injury or falls followed by road traffic accidents. Eight studies reported the ISS of included patients [23, 24, 26, 28–31], with an overall average of 6, and a study average ISS ranging from 4 to 9. The percentage of patients with an ISS greater than 16 ranged from 5 to 14% across five studies [24, 26–29].

**Table 2:** Patient characteristics

Name and year	Sample size	Age	Gender (% female)	Mechanism of injury	Injury severity score	Mortality
Brown et al. [23]	438,828	Median 80, IQR 73–86	61%	Blunt 99%	Median 9 IQR 4–10	Overall 4.4%
Caterino et al. [24]	34,499 (15,664 before criteria, 18,835 after criteria)	Median 82, IQR 77–87	79%	Blunt 99%	Median 5 IQR 4–10 ISS > 15 14% ISS 10–15 14% ISS < 10 72%	Overall: 6.8% Before: 7.1% After: 6.6%
Cull et al. [25]	92,780 (derivation 13,125, validation 79,655)	Not reported	Not reported	Fall on same level only	Not reported	Not reported
Ichwan et al. [26]	33,379	Mean 82, SD 7	69%	Blunt 99%	Mean 8 SD 7 ISS > 15 13% ISS 10–15 14% ISS < 10 72%	Overall: 6.8%
Newgard et al. [27]	5,021	Mean 81.5	67%	Fall 83.2%, Motor Vehicle Collision 5.7%	Mean 6.6 ISS > 15: 5%	Overall: 4.7%
Newgard et al. [28]	33,298 (derivation 19,897, validation 13,401)	65–74 years: 25.4%; 75–84 years: 37.8%; > 85 years: 36.8%	68%	Fall 79.6%, Motor Vehicle Collision 9.9%	0–8: 74%; 9–15: 21.5%; > 15: 4.5%	Overall: 3% ISS > 15: 18.7%
Newgard et al. [29]	44,890 (derivation 27,086, validation 17,804)	Median 77, IQR 64–85	63%	Fall 71.4%, Motor Vehicle Collision 16.5%	> 15: 2328 (5.2%) For ISS > 15 group: Median ISS 20 (IQR 17–26)	Overall: 2.7%
Nishijima et al. [30]	2,110	Median 73, IQR 62–85	40%	Head injury only Fall from standing or less 68%, fall greater than standing 3.8%, Motor Vehicle Collision 14.3%	Median 5 IQR (2–10)	Death in the ED: 0.1% Composite outcome of death or neurosurgery: 1.9%
Scheetz [31]	556,898	Mean (SD): 2004: 65.2 (9.2); 2007: 68.1 (9.9), 2008: 66 (9.1)	2004: 46.9%; 2007: 53.9%; 2008: 54.8%	Motor Vehicle Collision only	Mean (SD): 2004: 3.8 (6.2); 2007: 4.0 (7.3); 2008: 4.2 (6.9)	AIS 3 to 5: 2004: 1.2% 2007: 1.6% 2008: 1.4%
Wasserman et al. [32]	N/A	N/A	N/A	Traumatic brain injury	N/A	N/A
Werman et al. [33]	90,597	Not reported	Not reported	Not reported	Not reported	Single body region injuries: 3.9% Multiple body regions injuries: 8.4%

### Triage criteria

Eight distinct elderly triage criteria were reported, summarised in Table 3. Four studies derived triage criteria from previously collected patient records [25, 28, 29, 33]. The most commonly reported elderly triage criteria was the Ohio Geriatric Trauma Triage Criteria [4, 24, 33]. This was developed by Werman et al*.* [33] retrospectively applied to a trauma registry by Ichwan et al*.* [26] and its real-world application assessed by Caterino et al*.* using a retrospective interrupted time series [24]. All other triage criteria were reported in individual studies only [23, 25, 27–31]. One study developed binary regression equations of vital signs for each MOI [25].

**Table 3:** Elderly-specific triage criteria descriptions

	Elderly-specific trauma triage criteria	Modifications from corresponding adult criteria
Ohio Geriatric Trauma Triage Criteria Caterino et al. [24], Ichwan et al. [26], Werman et al. [33]	Physiologic: SBP < 100 or absent radial with carotid pulse present, GCS 14 or less in trauma with known to suspected TBI Anatomical: Fracture of 1 proximal long bone by MVC, injury sustained in 2 or more body regions Mechanical: pedestrian struck by motor vehicle, fall from any height, including standing falls, with evidence of TBI (decreased LoC from baseline, blurred vision, severe or persistent headache, nausea or vomiting, change in neurologic status, unequal pupils)	Ohio Adult Triage Criteria SBP changed from < 90 mmHg to < 100 mmHg GCS changed from 13 to 14 Change of long bone fracture number from 2 to 1 Inclusion of involvement in any motor vehicle accident Inclusion of fall from any height
Step 1 & 2 of 2011 Field Triage Scheme with SBP < 110 Brown et al. [23]	Step 1 Physiologic: GCS score ≤ 13, SBP < 110 mm Hg, RR < 10 or RR > 29 Step 2 Anatomical: penetrating injury, flail chest, open skull fracture, ≥ 2 proximal long bone fractures, pelvic fracture, crush injury, amputation, paralysis	2011 Field Triage Scheme (ACS-COT) SBP changed from < 90 mmHg to < 110 mmHg
Cull et al. [25]	Binary regression equation for vital signs: SBP, HR, RR, GCS. Specific equations for fall, cut/piercing injury, and gunshot wound	N/A
2011 Field Triage Scheme with anti-coagulant and anti-platelet Nishijima et al. [30]	Step 1 Physiologic: GCS score ≤ 13, SBP < 90 mm Hg, RR < 10 or RR > 29 Step 2 Anatomical: penetrating injury, flail chest, open or depressed skull fracture, ≥ 2 proximal long bone fractures, pelvic fracture, amputation proximal to wrist and ankle, paralysis, crushed degloved or mangled extremity Step 3 Mechanism: Fall greater than 20ft, high risk auto crash (intrusion > 12 inches at occupant site or > 18inches at any site, ejection, death in same compartment, vehicle telemetry consistent with high risk of injury), Auto vs pedestrian/bicycle thrown, run over, or > 20mph impact, motorcycle crash > 20mph Step 4 Special considerations: Older adults (risk of injury/death increases after age 55, SBP < 110 might represent shock after age 65, low impact mechanisms (e.g. ground level falls) might result in severe injury), anticoagulants and bleeding disorders, pregnancy > 20 weeks, EMS provider judgment OR anti-coagulant or anti-platelet use with head injury	2011 Field Triage Scheme (ACS-COT) Addition of anti-coagulant or anti-platelet use with head injury
Newgard et al. [27]	Current field triage (2011 ACS-COT) and if not met proceed to geriatric-specific physiological signs (GCS 14 or less, SBP ≤ 110 or ≥ 200, RR ≤ 10 or ≥ 24, HR ≤ 60 or ≥ 110) and if not met proceed to 2 or more co-morbidities	2011 Field Triage Scheme (ACS-COT) SBP changed from < 90 mmHg to < 110 mmHg or > 200 mmHg GCS changed from 13 to 14 RR changed from < 10 or > 29 to < 10 or > 24 HR criteria added Addition of ≥ 2 co-morbidities
Alternative triage guidelines Newgard et al. [28]	GCS 14 or less, SBP ≤ 110 or ≥ 200, RR ≤ 10 or ≥ 24, HR ≤ 60 or ≥ 110	2011 Field Triage Scheme (ACS-COT) SBP changed from < 90 mmHg to < 110 mmHg or > 200 mmHg GCS changed from 13 to 14 RR changed from < 10 or > 29 to < 10 or > 24 HR criteria added
Newgard et al. [29]	GCS 14 or less, SBP < 110 or > 200, RR < 10 or > 24 or need for assisted ventilation, shock index > 1.0	2011 Field Triage Scheme (ACS-COT) SBP changed from < 90 mmHg to < 110 mmHg or > 200 mmHg GCS changed from 13 to 14 RR changed from < 10 or > 29 to < 10 or > 24 Addition of shock index > 1.0
2006 Field Triage Scheme Scheetz [31]	Step 1 Physiologic: GCS score ≤ 13, SBP < 90 mm Hg, RR < 10 or RR > 29 Step 2 Anatomical: penetrating injury, flail chest, open or depressed skull fracture, ≥ 2 proximal long bone fractures, pelvic fracture, amputation proximal to wrist and ankle, paralysis, crushed degloved or mangled extremity Step 3 Mechanism: Fall greater than 20ft, high risk auto crash (intrusion > 12 inches at occupant site or > 18inches at any site, ejection, death in same compartment, vehicle telemetry consistent with high risk of injury), Auto vs pedestrian/bicycle thrown, run over, or > 20mph impact, motorcycle crash > 20mph Step 4 Special considerations – Age (older adults risk of injury/death increases after age 55), anticoagulation and bleeding disorders, time sensitive extremity injury, end-stage renal disease requiring dialysis, pregnancy > 20 weeks, EMS provider judgment	1999 Field Triage Scheme (ACS-COT) Addition of anticoagulation and bleeding disorders Addition of end-stage renal failure on dialysis Deletion of cardiac disease, respiratory disease, insulin-dependent diabetes mellitus, cirrhosis, morbid obesity, immunosuppression

Wasserman excluded as only applied to TBI (not alert or alert and not responding to commands warrants triage)

^*^ACS-COT = American College of Surgeons Committee on Trauma

All criteria included physiological parameters, and most included anatomical and MOI factors [23, 24, 26, 27, 30, 31, 33]. There was variability across all physiological parameter thresholds. Assessment of systolic blood pressure (SBP) was used in all triage criteria, with five using < 110 mmHg [23, 27–30], one < 100 mmHg [24, 26, 33], and two < 90 mmHg [30, 31]. One placed < 110 mmHg within a special considerations step and encouraged EMS judgment [30]. GCS thresholds were either ≤ 14/15 [24, 26–29, 33] or ≤ 13/15 [23, 30, 31]. The consensus panel used by Wasserman et al*.* focused on traumatic brain injury and concluded that pre-hospital GCS assessment was unreliable and recommended triage if unable to obey motor commands or not alert [32]. Heart rate thresholds were only used by two studies with values of ≤ 60 or ≥ 110 [27, 29]. Anatomical and MOI factors most commonly included head injury [24, 26, 30, 33], pedestrian struck by motor vehicle [24, 26, 30, 31, 33], and long bone or pelvic fractures [23, 24, 26, 29, 30, 33]. Two triage criteria placed emphasis on anticoagulation medication, particularly in the context of head injury [30, 31].

Most studies made modifications to existing adult triage criteria to produce their elderly-specific criteria (Table 3) [23, 24, 26–31, 33]. The majority used the Trauma Field Triage Scheme [23, 27–31] with the remainder using the Ohio Adult Triage Criteria [24, 26, 33]. The most common modification was an increase in the SBP threshold from < 90 mmHg to < 100 mmHg [24, 26, 33], or < 110 mmHg [23, 27–29]. GCS thresholds were increased from ≤ 13 to ≤ 14 [24, 26–29, 33]. Two studies also made changes to the respiratory rate parameters from < 10 or > 29 to ≤ 10 or ≥ 24 and added a heart rate criterion of either ≤ 60 bpm or ≥ 110 bpm [27, 28]. The Ohio Geriatric Trauma Triage Criteria added MOI criteria and included involvement in any motor vehicle accident and falls from any height [24, 26, 33]. This tool also altered anatomical criteria by reducing the number of suspected fractured bones from two to one [25, 29]. No other triage criteria made modifications to anatomical or MOI factors. Two studies added criteria to recognise anti-coagulant or anti-platelet use [30, 31], whilst another added two or more co-morbidities [27].

### Reported efficacy—sensitivity and specificity

Most studies used ISS as the gold standard to determine triage efficacy [23, 25–29, 31], with ISS > 15 in five studies [23, 26–29], and one used ISS ≥ 15 [25]. Otherwise overall Abbreviated Injury Score ≥ 3 [31] or, in patients with head injury, intracranial haemorrhage on initial CT head were used [30]. No study reported rates of triage of elderly patients with ISS > 9.

Eight studies reported triage criteria sensitivity and specificity, or under- and over-triage rates (Table 4) [23, 25–31]. Sensitivity was the proportion of positives, as per the study’s gold standard, correctly identified and specificity was the proportion of negatives correctly identified. One study reported under- and over-triage rates and this was converted to sensitivity and specificity for comparison [25]. Sensitivity ranged from 44% to 96.5% (median 88.8%) and specificity was 16.9% to 71% (median 51.5%) [23, 25–31]. One study found that adding geriatric-specific vital signs produced a sensitivity and specificity of 74.4% and 47.5%, compared with 39.9% and 89.9% for standard adult triage criteria [27]. Further addition of ≥ 2 comorbidities gave sensitivity and specificity of 91.3% and 16.9%, and subsequent addition of anti-coagulant use produced 95.6% and 14.0% [27]. The authors determined anti-coagulant use was not a good predictor and therefore excluded it from the final triage criteria. All studies comparing elderly-specific criteria with adult criteria found an increase in sensitivity with a decrease in specificity for elderly-specific criteria [23, 26–29]. This increase in sensitivity ranged from 4% to 26.9%, and the decrease in specificity varied from 4 to 50% [23, 26–29].

**Table 4:** Reported elderly-specific triage criteria efficacy

Study	Triage criteria	Definition of requiring triage	Triage criteria sensitivity	Triage criteria specificity
Brown et al. [23]	Step 1 & 2 of 2011 Field Triage Scheme with SBP < 110	ISS > 15	44% (40% if use SBP < 90)	71% (75% if use SBP < 90)
Caterino et al. [24]	Ohio Geriatric Trauma Triage Criteria	N/A	N/A	N/A
Cull et al. [25]	Binary regression equations of vital signs (HR, RR, SBP, GCS)	ISS ≥ 15	96.5%	37.1%
Ichwan et al. [26]	Ohio Geriatric Trauma Triage Criteria	ISS > 15	93% (CI 92–93%)	49% (CI 48–49%)
Newgard et al. [27]	Addition of geriatric-specific vital signs and ≥ 2 co-morbidities to current field triage	ISS > 15	91.3%	16.9%
Newgard et al. [28]	Alternative triage guidelines	ISS > 15	92.1% (CI 89.6–94.1%)	41.5% (CI 40.6–42.4%)
Newgard et al. [29]	GCS 14 or less, RR < 10 or > 24 or need for assisted ventilation, SBP < 110 or > 200, shock index > 1.0	ISS > 15	86.3%	60.7%
Nishijima et al. [30]	2011 Field Triage Scheme with anti-coagulant and anti-platelet	Intracranial haemorrhage on initial CT	59.5% (CI 42.9–74.2%)	67.2% (CI 61.1–72.7%)
Scheetz [31]	2006 ACS-COT Field Triage Decision Scheme. 2004 pre revision	AIS ≥ 3	2004 58% 2007 65% 2008 78%	2004 54% 2007 56% 2008 48%
Wasserman et al. [32]	Not alert or alert but does not obey commands	Would likely benefit from triage to a trauma centre	N/A	N/A
Werman et al. [33]	Ohio Geriatric Trauma Triage Criteria	Criteria associated with increased mortality risk in elderly patients	N/A	N/A

One study reported an increase in patients meeting criteria for triage to a trauma centre (TC) after adoption of new elderly-specific triage criteria (58% vs 44%, 13–15%, *p* < 0.001), yet only a 1% increase in transport of patients to the TC (0.1–2.0%, *p* < 0.05) [24].

### Clinical outcomes

Mortality was the only reported clinical outcome. Predetermined outcomes for investigation such as rates of interhospital transfer and life-saving interventions were not reported. Only one real-world study assessed the effect implementation of elderly-specific triage criteria had on mortality [24].

Most studies reported overall mortality, ranging from 2.7 to 6.8% (median 4.6%) [23, 24, 26–29]. Others reported mortality by Abbreviated Injury Score (AIS > 3 1.4%) [31], anatomy of injury (single body region 3.9%, multiple body regions 8.4%) [33], or a composite outcome of in-hospital death or neurosurgery (1.9%) [30]. One study assessed the efficacy of triage criteria to predict 30-day and 1-year mortality, in addition to ISS > 15 [27]. Sensitivity for 30-day and 1-year mortality were 94.4% and 93.5%, and specificity were 17.0% and 19.1% [27]. The sensitivity for the corresponding adult triage criteria on 30-day mortality and 1-year mortality were substantially different at 15.7% and 11.3%, as were specificity at 88.6% and 88.3% [27]. Only one study assessed mortality before and after implementation of elderly-specific triage criteria and found that unadjusted mortality was insignificantly reduced from 7.1 to 6.6% (*p* = 0.1) [24]. In adjusted analyses, mortality was significantly reduced in those with an ISS < 10 (OR = 0.81, 0.70–0.95, *p* = 0.01) and discharge to home was increased (OR = 1.06, 1.01–1.10, *p* = 0.02) [24]. The presence of co-morbidities in elderly patients had a greater impact on mortality than in non-elderly patients (pulmonary disease OR = 2.60, 2.11–3.20, *p* < 0.0001; clotting disorder OR = 1.99, 1.41–2.82, *p* < 0.0001; diabetes OR = 1.62, 1.37–1.91, *p* < 0.0001; coronary artery disease OR = 1.43, 1.18–1.73, *p* = 0.00015) [33], yet no other studies adjusted for co-morbidities or frailty in their sample [23–32].

### Risk of bias and quality assessments

There was variability in the risk of bias (Additional file 2). The highest scores were observed in two interrupted time series studies, however risk of confounding remained despite adjusted analyses [24, 31]. Lack of cohort demographics reporting prevented assessment of sample representativeness [25, 33]. The quality of evidence according to the GRADE system was “low” [23–29, 31, 33] or “very low” [30, 32] for all included studies (Additional file 3).

## Discussion

Eight unique elderly-specific criteria for pre-hospital triage following trauma are described and elderly age cut-offs varied from 55 to 70 years. Studies lacked consistency in triage criteria and physiological parameter adaptations for elderly patients. Sensitivity was varied but was improved when compared to corresponding adult triage criteria. Specificity was poor at less than 50% in over half of studies, therefore risking high rates of over-triage. In-hospital mortality was low, which may reflect the low severity of injury across the studies. There was a lack of evaluation of clinical outcomes in real-world application of elderly-specific triage criteria. This review illustrates the uncertainty in how best to identify and triage elderly trauma patients and it remains unclear which elderly trauma triage criteria is superior.

Variation in age cut-offs reflects the continued uncertainty in defining the elderly trauma population [34]. Four of the eleven studies used a cut-off of 55 years, a threshold that would be not considered elderly in many healthcare systems [34, 35]. Risk of adverse outcome following trauma increases with age [34, 36], therefore although a dichotomous age cut-off is convenient, it may be inappropriate. Age-based markers may support a more nuanced approach [37], however no tools utilised these. These may be useful in development of elderly-specific triage tools and are an avenue for research [37]. Furthermore, there is an increasing recognition that frailty and sarcopenia are associated with adverse outcome following trauma [38, 39]. Indeed frailty assessments appear superior to age in predicting outcome [40], however no published triage tools utilised frailty scoring. Co-morbidities appear to increase risk of adverse outcome, although current evidence is inconsistent [5, 33, 34, 36, 41]. Nevertheless, only one tool included co-morbidity assessment using a crude ≥ 2 co-morbidities [27]. Specific pre-existing conditions and their severity may adversely impact outcome and a more refined assessment could be beneficial [34, 42]. This review illustrates a growing consensus for elderly-specific triage criteria to include adapted physiological parameters, most commonly by increasing SBP and GCS thresholds. This reflects the altered physiological response to injury and the increased morbidity associated with higher values than in adult patients [15]. Significant traumatic injuries frequently occur in elderly patients after low energy mechanisms [4, 5], however only one tool included fall from any height as a component. There is a necessity for tools to be developed in line with the evolving knowledge of how major trauma occurs in elderly patients. Similarly, local guidance and EMS education should be informed by this developing understanding, which may also support improvements in care. The discordance found by Caterino et al*.* in elderly patients meeting elderly-specific triage criteria and actual transportation rates underlines this importance of continued evidence-based training and education [24]. Triage tools must be accessible to EMS teams in a fast-paced and often austere pre-hospital environment. Most elderly-specific triage criteria were modifications of existing adult field triage tools and adaptations in this way may best support tool implementation [43].

The under-triage of elderly trauma patients and associated harm is well-reported, and improving sensitivity of elderly trauma triage is a priority [5, 11, 13, 44]. The published elderly-specific triage criteria substantially improve upon adult triage tools, although the reported sensitivity is highly variable. This comes with a reduction in specificity. The elderly trauma population is substantial and growing, therefore high rates of over-triage would place significant demand on services. Pre-hospital identification of elderly patients suffering significant traumatic injuries may be used to inform more than just transport location. Trauma units may be the most appropriate location of care and offer multiple advantages over major trauma centres for elderly patients [45], hence measuring triage in terms of transportation location may be overly simplistic. Pre-hospital identification of those at high risk of adverse outcome may direct in-hospital care such as differential levels of trauma activation, earlier imaging and senior clinician assessment, use of multi-disciplinary elderly trauma teams, inform hospital care pathways, and support staff guidance and education [46, 47]. This has the potential to use finite resources in a more precise and targeted manner. Studies used ISS > 15 as the gold standard to assess sensitivity and specificity. Whilst this is the traditional definition of major trauma, this may be an imperfect and inappropriate gold standard in elderly patients [48]. ISS is unable to account for the impact of frailty, multiple injuries to one body area, and may not reliably predict the need for life-saving interventions, hence its validity as an arbitrary definition of major trauma is challenged [49, 50]. Moreover, adverse outcome may occur at lower ISS in elderly patients and therefore identification of patients with lower ISS may be needed [51]. This review also intended to evaluate criteria efficacy in terms of ISS > 9, along with a range of pre-determined clinical outcomes in addition to mortality, including delivery of life-saving interventions, however no study reported these outcomes. This demonstrates the need for future studies to better understand the effect of elderly trauma triage tools on a range of clinical and system-related outcomes.

The average ISS of this review’s included studies reflects its included population of all elderly trauma patients. Some reports have only included patients with an ISS > 15 [5]. A broader inclusion is important to allow assessment of the effects of elderly-specific triage criteria across the whole elderly trauma population. The widely reported high under-triage rates of elderly trauma patients highlights the difficulties in identifying these patients [5, 11, 13, 14, 24, 44]. A clinically implemented elderly-specific triage tool will therefore need to apply to the whole elderly trauma population in order to reduce this substantial under-triage rate.

The low mortality of included studies likely reflects the injury severity. The review reveals the scarcity of real-world outcome data concerning elderly-specific triage criteria. Caterino et al*.* showed a reduction in mortality after criteria adoption, however despite adjusted analysis, residual confounding is likely to remain with this study design [24]. There was minimal change in transportation rates and so improvements in outcome may reflect other changes in care rather than direct effects of triage criteria adoption [24]. Werman et al*.* was the only study to concurrently assess predictors of mortality and these were concordant with published literature [33, 34]. There is a need to assess the effect of elderly-specific criteria on mortality and other sensitive measures of recovery within other geographical populations, in order to improve outcomes for this vulnerable, high-risk group [5].

The principal limitation of this systematic review is the risk of bias and low quality of the included studies. All studies were USA-based therefore it is unclear how translatable this data is to other systems and transportation geographies. Finally, this systematic review was limited to published data and we were unable to capture local EMS elderly-specific triage criteria not reported in the current literature [52]. Publication and peer-review of all elderly-specific triage criteria are needed to support an evidence-based approach to improve care.

## Conclusions

To date, this is the only systematic review of pre-hospital elderly trauma triage criteria and includes over 1.3 million patient records from eleven studies. There are multiple published elderly-specific criteria for pre-hospital trauma triage, all of which were developed from USA-based populations. The age cut-off to define the elderly trauma patient was inconsistent, as were the criteria components, which were generally adaptations of adult triage criteria. There appears to be a developing consensus regarding the inclusion of higher thresholds for physiological parameters, namely SBP and GCS. There is a paucity of real-world data evaluating the impact of elderly-specific triage criteria on clinical outcomes. Measurement and subsequent publication of clinically employed elderly-specific criteria is required to inform practice and support improvements in clinical care and outcomes for this growing and vulnerable population.

## Supplementary Information

Additional file 1. Screenshot of search.

Additional file 2. Newcastle Ottawa Risk of Bias.

Additional file 3. GRADE quality of evidence assessment.

## Data Availability

Systematic review of published literature with references to all including studies. The datasets used and/or analysed during the current study are available from the corresponding author on reasonable request.

## Abbreviations

AIS: Abbreviated injury score
EMS: Emergency medical services
GCS: Glasgow coma scale
GRADE: Grading of Recommendations, Assessment, Development, and Evaluation
ISS: Injury severity score
MOI: Mechanism of injury
OR: Odds ratio
SBP: Systolic blood pressure
