# Supplementary figures and images for: Evaluation of elderly specific pre-hospital trauma triage criteria: a systematic review
Source: Scand J Trauma Resusc Emerg Med. 2021 Aug 30;29:127. doi: 10.1186/s13049-021-00940-z (PMC8404299; doi:10.1186/s13049-021-00940-z)

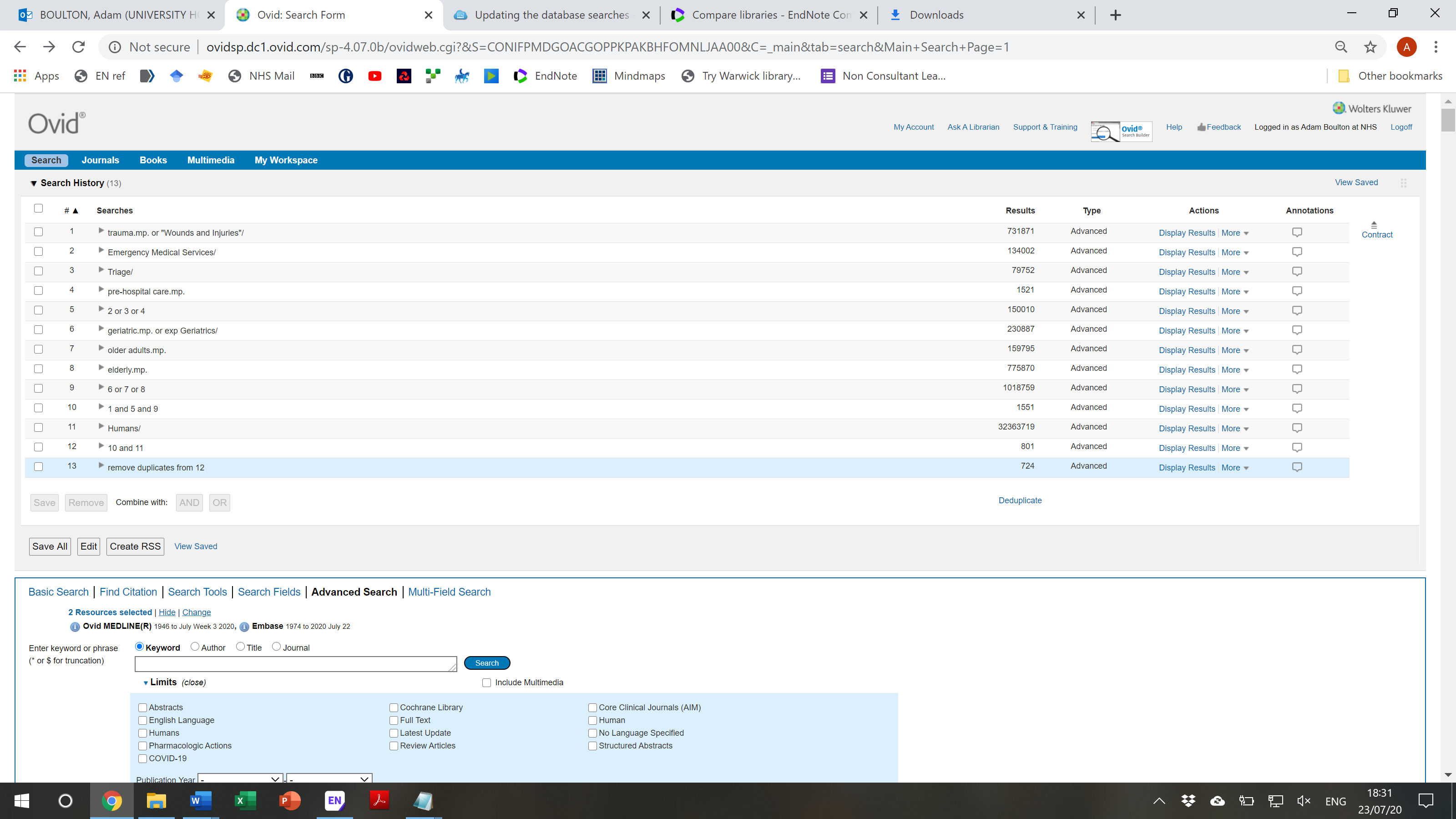


**Supplementary File 1** Screenshot of search

Supplement: Supplementary file 1 — Screenshot of search. [file 13049_2021_940_MOESM1_ESM.docx]
