# Supplementary material for: Evaluation of elderly specific pre-hospital trauma triage criteria: a systematic review
Source: Scand J Trauma Resusc Emerg Med. 2021 Aug 30;29:127. doi: 10.1186/s13049-021-00940-z (PMC8404299; doi:10.1186/s13049-021-00940-z)
Supplement: Supplementary file 2 — Newcastle Ottawa Risk of Bias. [file 13049_2021_940_MOESM2_ESM.docx]

| **STUDY** | | **SELECTION** | | | | **COMPARABILITY** | | **OUTCOME** | | | **SCORE** |
| --- | --- | --- | --- | --- | --- | --- | --- | --- | --- | --- | --- |
| Author | Year | **Representativeness** | | **Exposure** | **Outcome** | Controls for | | Assessment | Follow up | |  |
|  |  | Exposed | Non-exposed | Ascertainment of exposure | Outcome not previously present | Most important | Other factor |  | Length | Adequacy |  |
| Brown | 2015 | * | . | * | * | . | . | * | * | * | 6 |
| Caterino | 2016 | * | * | * | * | * | . | * | * | * | 8 |
| Cull | 2019 | . | . | * | * | . | . | * | * | * | 5 |
| Ichwan | 2015 | * | . | * | * | . | . | * | * | * | 6 |
| Newgard | 2019 | * | * | * | * | . | . | * | * | * | 7 |
| Newgard | 2016 | * | * | * | * | . | . | * | * | * | 7 |
| Newgard | 2014 | * | * | * | * | . | . | * | * | * | 7 |
| Nishijima | 2017 | * | . | . | * | . | . | * | * | * | 5 |
| Scheetz | 2011 | * | * | * | * | * | . | * | * | * | 8 |
| Werman | 2011 | . | . | * | * | . | . | * | * | * | 5 |

**Supplementary File 2** Newcastle Ottawa Risk of Bias
