# Supplementary material for: Evaluation of elderly specific pre-hospital trauma triage criteria: a systematic review
Source: Scand J Trauma Resusc Emerg Med. 2021 Aug 30;29:127. doi: 10.1186/s13049-021-00940-z (PMC8404299; doi:10.1186/s13049-021-00940-z)
Supplement: Supplementary file 3 — GRADE quality of evidence assessment. [file 13049_2021_940_MOESM3_ESM.docx]

| **Study** | **Design** | **Reason to upgrade or downgrade** | **Overall assessment** |
| --- | --- | --- | --- |
| Brown 2015 | Non-RCT (Low) | Nil | ⊕⊕⊝⊝ Low |
| Caterino 2016 | Non-RCT (Low) | Nil | ⊕⊕⊝⊝ Low |
| Cull 2019 | Non-RCT (Low) | Nil | ⊕⊕⊝⊝ Low |
| Ichwan 2015 | Non-RCT (Low) | Nil | ⊕⊕⊝⊝ Low |
| Newgard 2019 | Non-RCT (Low) | Nil | ⊕⊕⊝⊝ Low |
| Newgard 2016 | Non-RCT (Low) | Nil | ⊕⊕⊝⊝ Low |
| Newgard 2014 | Non-RCT (Low) | Nil | ⊕⊕⊝⊝ Low |
| Nishijima 2017 | Non-RCT (Low) | Risk of bias (-1) | ⊕⊝⊝⊝ Very Low |
| Scheetz 2011 | Non-RCT (Low) | Nil | ⊕⊕⊝⊝ Low |
| Wasserman 2015 | Non-RCT (Low) | Risk of bias (-1) | ⊕⊝⊝⊝ Very Low |
| Werman 2011 | Non-RCT (Low) | Nil | ⊕⊕⊝⊝ Low |

**Supplementary File 3** GRADE quality of evidence assessment
